# In Sunset Light

**Published:** 1881-12

**Authors:** K. E. Clark


[From the Home Journal ]

### IN SUNSET LIGHT.

I looked up, with childhood's laughing eyes,
To the brilliant sunset hues,
Nor thought but the changing tints were meant
My present to amuse.

In youth I scanned with earnest gaze
What the evening clouds might tell,
And I saw the wondrous future-land—
The path I knew full well.

In manhood's weary task I paused,
For the dust I scarce could see ;
Of childhood's joy, of a youthful dream,
The sunset spoke to me.

Through aged eyes I'm peering now,
No honors round me shine ;
Yet broader sight and calm content
Enrich my days' decline.

The splendor of the evening sky
Like music fills the air ;
Soft waves of golden melody
Seem pulsing everywhere.

I trace the subtle tie that binds
All beauties into one ;
And, in the sunset light, I know
My life has just begun.

K. E. Clark.

